# Effects of E-health-based interventions on glycemic control for patients with type 2 diabetes: a Bayesian network meta-analysis

**DOI:** 10.3389/fendo.2023.1068254

**Published:** 2023-05-04

**Authors:** Xiaoyue Zhang, Lanchao Zhang, Yuxin Lin, Yihua Liu, Xiaochen Yang, Wangnan Cao, Ying Ji, Chun Chang

**Affiliations:** Department of Social Medicine and Health Education, School of Public Health, Peking University Health Science Center, Beijing, China

**Keywords:** type 2 diabetes, E-health, glycemic control, Bayesian, network meta-analysis

## Abstract

The high disease burden of type 2 diabetes seriously affects the quality of life of patients, and with the deep integration of the Internet and healthcare, the application of electronic tools and information technology to has become a trend for disease management. The aim of this study was to evaluate the effectiveness of different forms and durations of E-health interventions in achieving glycemic control in type 2 diabetes patients. PubMed, Embase, Cochrane, and Clinical Trials.gov were searched for randomized controlled trials reporting different forms of E-health intervention for glycemic control in type 2 diabetes patients, including comprehensive measures (CM), smartphone applications (SA), phone calls (PC), short message service (SMS), websites (W), wearable devices (WD), and usual care. The inclusion criteria were as follows: (1) adults (age≥18) with type 2 diabetes mellitus; (2) intervention period ≥1 month; (3) outcome HbA1c (%); and (4) randomized control of E-health based approaches. Cochrane tools were used to assess the risk of bias. R 4.1.2 was used to conduct the Bayesian network meta-analysis. A total of 88 studies with 13,972 type 2 diabetes patients were included. Compared to the usual care group, the SMS-based intervention was superior in reducing HbA1c levels (mean difference (MD)-0.56, 95% confidence interval (CI): -0.82 to -0.31), followed by SA (MD-0.45, 95% CI: -0.61 to -0.30), CM (MD-0.41, 95% CI: -0.57 to -0.25), W (MD-0.39, 95% CI: -0.60 to -0.18) and PC (MD-0.32, 95% CI: -0.50 to -0.14) (p < 0.05). Subgroup analysis revealed that intervention durations of ≤6 months were most effective. All type of E-health based approaches can improve glycemic control in patients with type 2 diabetes. SMS is a high-frequency, low-barrier technology that achieves the best effect in lowering HbA1c, with ≤6 months being the optimal intervention duration.

**Systematic review registration:**

https://www.crd.york.ac.uk/prospero, identifier CRD42022299896.

## Highlights

We compared previous randomized controlled E-health interventions for self-management behaviors in patients with type 2 diabetes to determine which forms of interventions and what durations were most effective in lowering HbA1c levels.

We found that SMS is a high frequency, low cost, low barrier technology that achieves the best effect in lowering HbA1c, with an optimal duration of ≤6 months.

Future studies should fully investigate the advantages of various forms and combinations of intervention methods to maximize the effectiveness of disease management.

## 1 Introduction

The pervasiveness of unhealthy habits, including high-calorie intake and sedentary lifestyles, has increased the prevalence and lowered the age of onset of type 2 diabetes mellitus (1, 2). Research showed that the number of people with type 2 diabetes worldwide was approximately 462 million in 2017, with a prevalence of 6,059 cases per 100,000 (3). With the long course of the disease, symptoms such as thirst, frequent urination, and blurred vision continuously plague patients, resulting in increased negative emotions and difficulties (4). Cardiovascular disease is more likely to develop in patients with diabetes, and the risk rises as glycemic control deteriorates (5). Additionally, persons with diabetes are at a 2-4 times greater risk of death than adults without diabetes (6). Moreover, a study noted that 17.0% of type 2 diabetes patients reported moderate or severe depressive symptoms, and 10.6% suffered from major depressive disorder (7).

Glycemic control is the foundation of type 2 diabetes management; it can prevent cardiovascular disease-related and microvascular complications and impact patient survival (8). A Swedish study found that patients with HbA1c ≤6.9% (age under 55) had high risk of death , approximately two-fold higher than that in general population), while patients with HbA1c levels ≥9.7% (age under 55) had approximately four times higher risk (9). However, the glycemic control of type 2 diabetes patients is typically poor, and relevant studies have shown that fewer than 50% of type 2 diabetes patients have HbA1c below 7% (10).

Among the factors affecting glycemic control in patients, in addition to drug and insulin therapy, self-management (SM) is an essential aspect. The International Diabetes Federation emphasizes the need for patients' to manage their behavior, including blood glucose monitoring, medication compliance, diet control, and exercise management (11, 12). A meta-analysis revealed that SM behaviors such as physical exercise and regular blood glucose monitoring improve the quality of life of type 2 diabetes patients (13).

However, the SM behaviors of patients are generally poor, with one survey from China revealing that only 16.4% of such patients were able to maintain appropriate exercise and blood sugar monitoring, with most not meeting these standards (14). Ji M et al. showed that only 10% of 207 type 2 diabetes patients maintained an adequate diet and were more likely to have better glycemic control than others with a lower-quality diet (15). SM in patients with type 2 diabetes is long-term in nature and requires knowledge of the disease. Patients are often confronted with multiple obstacles, such as negative emotions associated with the long disease course, misconceptions about the disease, insufficient knowledge, financial burden, and work environment, that hinder the adoption of behaviors (16). For example, low medication adherence can affect the effectiveness of disease control because some patients believe that taking medications is addictive or refuse to take them because they consider doctors to be prescribed them for their own financial benefit; sedentary behavior and irregular working hours due to the nature of the occupation can also have an impact on the patient’s lifestyle, which in turn affects their disease management and glycemic control (17, 18).

The development of the internet and information technology has promoted innovation in disease management. E-Health was proposed at the beginning of the 21st century and has been widely used in diabetes management, including smartphone applications (SA), phone calls (PC), short message service (SMS) or tele-monitoring (19–25). Hansen demonstrated that type 2 diabetes patients who used E-health for disease management were more than twice as likely to make lifestyle changes than those using traditional methods (26). Numerous studies have confirmed the effectiveness of E-health interventions for glycemic control. A meta-analysis discovered that smartphone-based interventions for SM helped in reducinged HbA1c by approximately 0.51% compared to standard care; additionally, the researchers found that the shorter the duration of type 2 diabetes, the better the control (27). Tchero et al. found that telemedicine improved the treatment cooperation of patients and helped in controlling their glycemic status, reducing HbA1c by approximately 0.48% in patients with type 2 diabetes compared to the HbA1c levels of patients in standard care group (28).

Several recent studies have investigated the feasibility of using different types of E-health interventions, including PC, SMS, or SA, in the management of type 2 diabetes, but have not comprehensively explored which form of E-health intervention has a superior effect on glycemic control. Therefore, this study aims to use network meta-analysis to compare the differences and effectiveness of various types of E-health interventions in the disease management and glycemic control of patients.

## 2 Materials and methods

### 2.1 Study design

This study with network meta-analysis (NMA) was reported following the PRISMA statement (29) and the PRISMA-NMA statement extension for NMA (30). The protocol was registered in the PROSPERO International Prospective Register of Systematic Reviews (No:CRD42022299896).

### 2.2 Research question

The aim of this study is to evaluate the effectiveness of different forms of E-health interventions for- glycemic control in type 2 diabetes patients. The PICO related to this aim was as follows.

(1) Population: adults (age≥18) with type 2 diabetes mellitus
(2) Interventions: different forms of E-health for type 2 diabetes management, including SA, PC, SMS, websites (W), wearable devices (WD) and their combinations: comprehensive measures (CM).
(3) Comparisons: usual care (UC), including regular examinations, follow-up visits, and doctor-advised treatment options.
(4) Outcome: HbA1c, which has been widely used in many studies to reflect glycemic control level and intervention effectiveness (31, 32).

### 2.3 Data sources and search strategy

We searched three core databases (PubMed, Embase and Cochrane) and a clinical trial registration database (Clinical Trials.gov) from database inception until May 2022. Our search query is shown in **Supplementary Table 1**; the search were performed in July 2022. All studies included in the search were in English.

### 2.4 Inclusion and exclusion criteria

The inclusion criteria were as follows: (1) adults (age≥18) with type 2 diabetes mellitus; (2) minimum follow-up duration of 1 month; (3) HbA1c (%) outcomes; and (4) randomized controlled trials (RCTs) of E-heath interventions.

Studies were excluded if (1) patients had serious complications or mental disorders; (2) patients were considered to have gestational diabetes or type 1 diabetes mellitus; (3) outcome values in the study were insufficient to perform NMA; (4) non-RCT studies, such as protocols, conference abstracts, or systematic reviews; and (5) the results of the study could not be found.

### 2.5 Data extraction

All the identified studies were exported to Endnote X9. Two researchers, with Master’s in Public Health and well-trained for NMA before the study, reviewed study titles and abstracts after removing duplicates and independently marked them for inclusion or exclusion. They downloaded and read the full text of all remaining articles for full-text screening and data collection. In this step, data included year and country of publication, authors, description of intervention and control, HbA1c, sociodemographic characteristics (gender, age, and race), sample size, intervention period, and follow-up duration.

In addition, to summarize key points about different forms of interventions, researchers collected and assessed details of interventions, including intervention strategies (behavior reminder, information feedback, health education course, blood glucose data monitoring, SM guidance and encouragement and emotional support), frequency of intervention (high, middle, low or unclear), health provider involvement (yes or no), interactivity (yes or no), and personalization (yes or no). Their criteria were as follows.

Intervention strategies: We analsis guiding and training methods to improve the behavior of patients through E-health interventions and define intervention strategies as behavior reminder, information feedback, health education, blood glucose monitoring, SM guidance and encouragement and emotional support. Behavior reminder—the reminders such as patient medication reminders and urging patients to exercise and maintain a healthy diet were considered in; information feedback—whether a patient could get feedback related to their own situation during the intervention, for example, receiving the blood glucose curve from the monitoring instruments, apps, or a message; health education—researchers used health education course, including video and live classes, to intervene in patient's behavior; blood glucose monitoring—patients can apply E-health devices to record their blood glucose data and can view their blood glucose values and changes; SM guidance—guidance regarding a reasonable diet, proper exercise, timely medication, and blood glucose monitoring; encouragement and emotional support—addressing the psychological obstacles faced by patients through companionship, talking to people, or using psychotherapeutic strategies to alleviate their negative emotions and enhance disease management.

Frequency: High frequency was defined as intervention more frequent than once a week, middle frequency - once a week to once a month, and low frequency – less than once a month.

Health provider involvement (Yes): If health care personnel (including doctors, nurses, nutritionists, etc.) participated in the intervention process. Whether an article showed health provider involvement was determined by our judgement.

Interactivity (Yes): Whether bidirectional informative feedback exists is the primary principle for judging interactivity. If the researchers and patients gave information in the form of E-health platforms and communicated with each other during the intervention, it can be defined as interactivity. The offline face-to-face instructions regarding the study, including instructions on device use before the start of the study or instructions from the physician during the routine treatment of patient’s, cannot be counted as interactivity, and we define interactivity primarily as the part conducted using E-health platforms.

Personalization (Yes): If the research team could provide individualized interventions or adjust the intervention strategies according to the patient’s conditions, we judged that the intervention described in the article was personalized.

### 2.6 Definition of different forms of E-health

With reference to the definition of E-health (19, 20) and previous studies that divided E-health interventions into five forms (33), we summarized the forms of E-health intervention into the following six categories: SA, PC, SMS, W, WD, and their combinations coded as comprehensive measures (CM). Their definitions in this study are as follows.

- W: interventions based on websites, which are widely used in computer-based interventions.
- PCs: interventions based on phone calls.
- SMSs: interventions based on short messages, which are basic functions of smartphones.
- WDs: interventions based on wearable devices that measure and upload biological data, including mobile blood glucose detectors, heart rate monitors, and pedometers.
- SA: interventions based on smartphone applications, including those involved in making voice or video calls, sending short messages or feedback, and collecting biological data.
- Interventions including two or more forms of E-health platforms were defined as CM.

W are interventions based on websites, which widely used in computer-based interventions. PC are interventions based on phone calls while SMS are interventions based on short messages, which are both basic functions of the smartphone. WD are interventions based on wearable devices that measure and upload biological data, including mobile blood glucose detectors, heart rate monitors, and pedometers. SA are interventions based on smartphone applications, including those involved in making voice or video calls, sending short messages or feedback, and collecting biological data. Interventions including two or more forms of E-health were defined as CM.

### 2.7 Assessment of bias and overall quality of evidence

Using Cochrane Collaboration’s risk of bias tool (34), two authors independently assessed all studies for bias in (1) random sequence generation, (2) allocation concealment, (3) blinding of participants and personnel, (4) blinding of outcome assessment, (5) incomplete outcome data, (6) selective reporting and (7) other. Each bias was marked as low, unclear, or high. Both authors discussed any disagreements and consulted the third author if needed. Publication bias was evaluated using Egger’s test (35) and visualized in a funnel plot.

### 2.8 Data analysis

RevMan (Version 5.4. The Cochrane Collaboration, 2020) was used for data analysis in the assessment of bias, while Stata (StataCorp. 2015. Stata Statistical Software: Release 15. College Station, TX) was used to perform Egger’s test and sensitivity analysis and draw a funnel plot.

R 4.1.2 (R Foundation for Statistical Computing, Vienna, Austria Package: gemtc) was used to conduct the Bayesian random-effects NMA, which can evaluate multiple interventions even if they were not directly compared in the studies.

We used nodes to represent different forms of interventions and edges to represent comparisons between interventions in the reticulated relationship plot (drawn by Stata 15.0 to achieve a better effect). As the primary outcome is HbA1c (%), which is a continuous variable with a normal distribution, the results are presented as mean difference (MD) and standard deviation (SD) for HbA1c with a 95% confidence interval (CI) and shown in network forest plots, where in lower values indicate better treatment. In addition, the league table of E-health intervention effects was used to summarize the direct and indirect comparison results, while the E-health intervention effect ranks were evaluated by their distribution of ranking probability and the surface under the cumulative ranking curve (SUCRA).

Based on the Bayesian Markov chain Monte Carlo algorithm, the results of the random effect model were evaluated and processed *a priori* (four chains were used for simulation analysis; the initial value was 2, the iteration times were adjusted by 20000, and the simulation iteration times were 50000). Study heterogeneity was evaluated using Higgins I2 values, which were <25%, 25~50%, and >50%, indicating low, moderate, and high heterogeneity, respectively. The node split model was used for the inconsistency test. If there was no statistical difference among the studies in the subgroup, the heterogeneity of the included studies was considered small, and the consistency model was used for analysis; otherwise, the inconsistency model was used for analysis.

In addition, subgroup analysis was performed according to gender, age, follow-up duration, and race to explore the causes of heterogeneity and inconsistency and differences in the effectiveness of interventions among different studies. Based on gender, age, and race, the patients were divided into two groups each, depending on whether the male proportion was above 50%, age was above 60 years, and race was white, respectively.

## 3 Results

**Figure 1:**
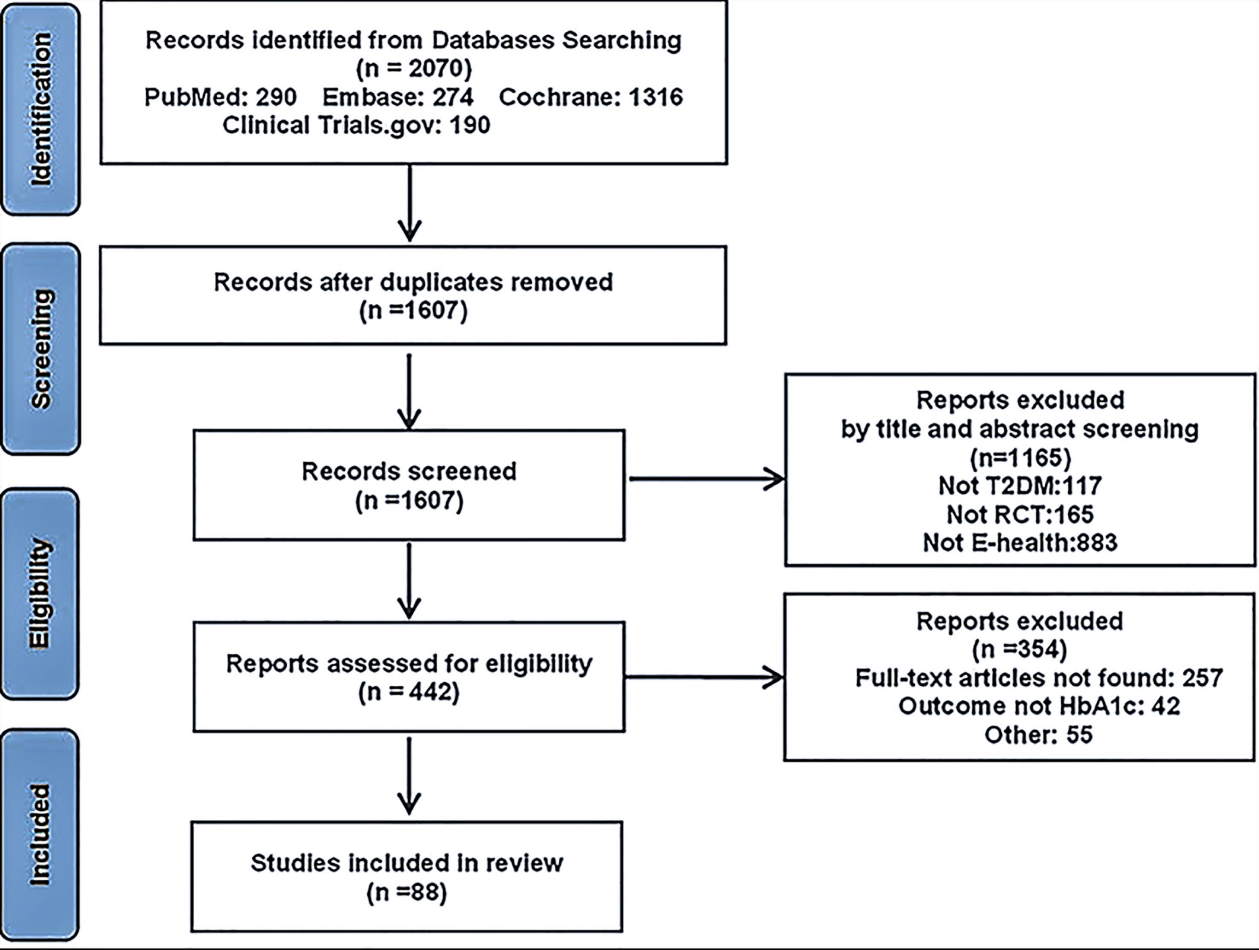
PRISMA flow chart of the study selection process.

Overall, 1880 and 190 records were identified from electronic databases and other sources, respectively. After duplicates were removed, the 1607 remaining records were screened based on the inclusion and exclusion criteria, among these 1165 records were excluded for the following reasons: other than type 2 diabetes (117), not RCT (165), not E-health, or not focusing on lifestyle modification (883); thus, full texts were sought for the remaining 442 records. Finally, 88 records remained after full-text screening. **Figure 1** shows the process of data extraction.

### 3.1 Characteristics of the included studies

A total of 88 studies with 13,972 type 2 diabetes patients were included. The study characteristics included country, author and publication year, intervention description, sample size, follow-up duration, age, and gender. These RCTs were conducted across 29 countries, with 17.0% from America, 15.9% from China, 10.2% from Korea, and 5.7% from Australia. The mean (SD) age of the patients was 57.6 (5.61) years; 55.1% were male, and 56.8% were white (**Table 1**). These studies were published between 2000 and 2022, and their follow-up period ranged from 1 to 24 months (**Supplementary Table 2**).

**Table 1:** Characteristics of participants and the studies (n = 88) included in network meta-analyses.

Characteristics		Value
Patients Baseline Characteristics(n=15,741)		
Age(mean, sd)		57.6 ± 5.61
Gender(male, %)		55.1%
Race(white, n %)		56.8%
Study Characteristics (n=13,972)		
Country(n,%)	US	15 (17.0%)
	China	14 (15.9%)
	Korea	9 (10.2%)
	Australia	5 (5.7%)
	UK	4 (4.5%)
	Spain	4 (4.5%)
	Other	37 (42.0%)
Continent	North America	17 (19.3%)
	Asia	40 (45.5%)
	Europe	25 (28.4%)
	North America	17 (19.3%)
	Oceania	5 (5.7%)
	Africa	1 (1.1%)
No of Arm	2	84
	3	4
Trial number of interventions	CM	22
	PC	17
	SA	27
	SMS	9
	W	13
	WD	10
	UC	84
Patients number of interventions	CM	1704
	PC	1305
	SA	1634
	SMS	1065
	W	882
	WD	894
	UC	6488

### 3.2 Assessment of bias

**Figure 2:**
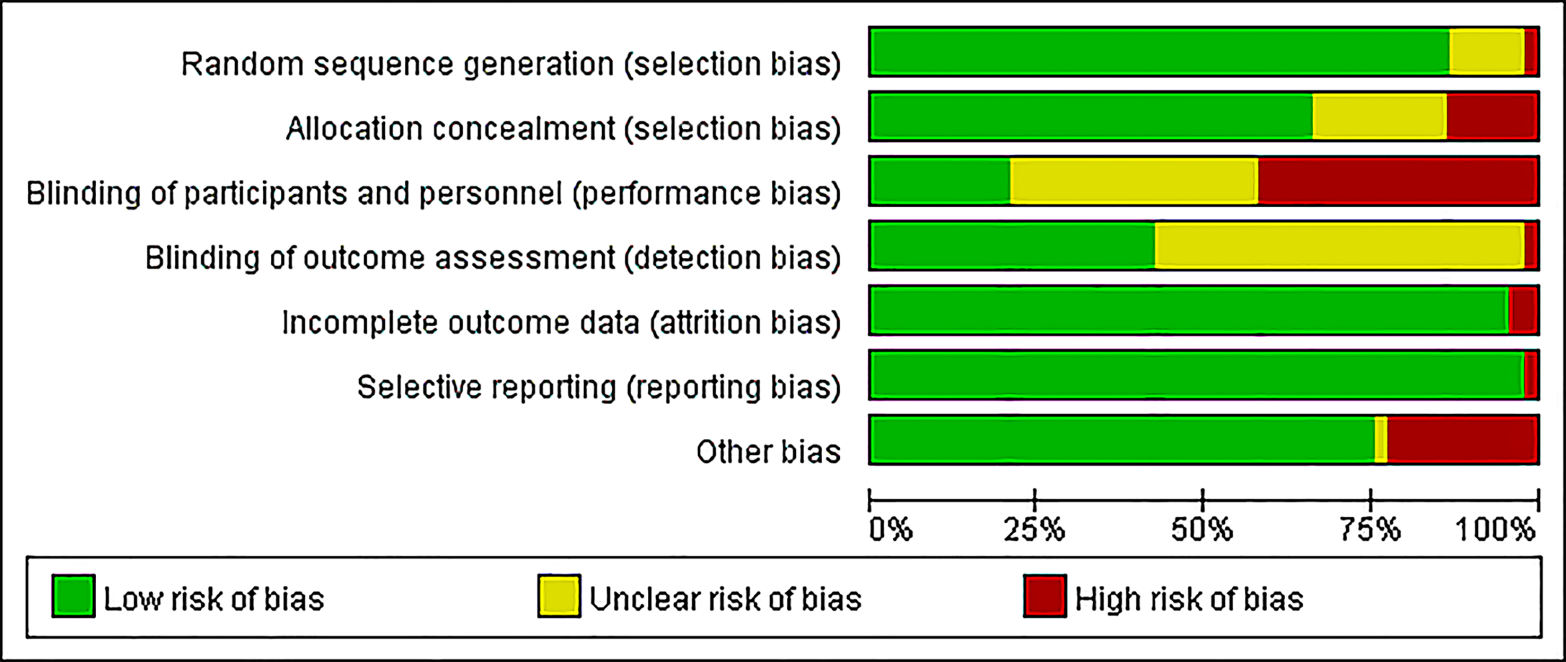
Risk of bias graph.

Assessment of bias was calculated using Cochrane Collaboration’s risk of bias tool (**Figure 2**). Most studies had a low risk of bias in random sequence generation (76, 86.4%), incomplete outcome data (84, 95.5%), selective reporting (86, 97.7%), and other biases (66, 75.0%). Several studies did not mention the methods for blinding; 33 (37.5%) studies had an unclear risk in both blinding of participants and personnel, while another 49 (55.7%) studies had an unclear risk only in the blinding of outcome assessments. Due to the interventions, it was difficult to implement the concealment and blinding of participants and personnel, which resulted in a high risk of bias (13.6% and 42.0%, respectively). Publication bias was judged using funnel plots, which were also plotted for each group of interventions and subjected to Egger tests with p values ranging from 0.062 to 0.876 (**Supplementary Tables 3-8**).

### 3.3 Outcomes

**Figure 3:**
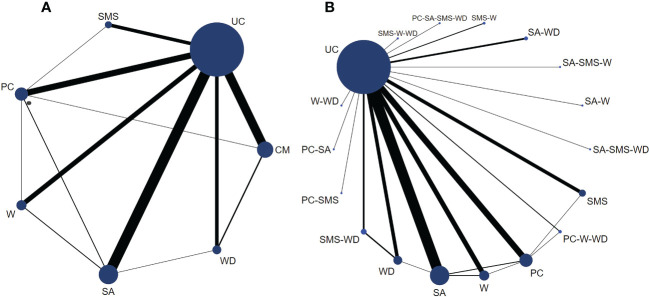
Network plots for HbA1c (%) control in type 2 diabetes patients based on E-health interventions. Legend: The size of the circle represents the number of patients receiving the interventions or control measure, and the width of the lines represents the number of studies. **(A)** is a network plot of six types of E-health interventions versus controls. **(B)** is a subset of comprehensive measures, which are a network plot of all intervention types and control groups included in the study.

A total of 88 randomized controlled trials of glycemic control using E-health in patients with type 2 diabetes were included in this study. These trials were mainly designed to compare the effect of different forms of E-health interventions (including CM, PC, SA, SMS, W, and WD) with those of UC (**Figure 3**).

PC-based intervention had the highest levels of health provider engagement, interactivity and personalization. Among all PC-based intervention trials, 88.2% trials showed health provider involvement, 94.1% trials demonstrated personalization, and all trials showed interactivity. CM, SMS and WD-based interventions performed better in terms of intervention frequency, and their constituent ratios of “high” frequency were 72.7%, 77.8% and 88.9%, respectively (**Table 2**).

**Table 2:** Main strategies and characteristics of E-health interventions.

Intervention	Content	HP	I	P	F	SUCRA	
		Yes%	Yes%	Yes%	High%	Rank	%
CM	1.Behavior Reminder 2.Information Feedback 3.Health Education 4.Disease Data Monitoring 5.SM Guidance	59.1%	63.6%	81.8%	72.7%	3	29.3%
PC	1.Behavior Reminder 2.Encouragement and Emotional Support 3.Health Education 4. SM Guidance	88.2%	100%	94.1%	50.0%	5	45.1%
SA	1.Behavior Reminder 2.Information Feedback 3.Health Education 4.Disease Data Monitoring 5. SM Guidance	59.3%	77.8%	63.0%	59.3%	2	37.6%
SMS	1.Behavior Reminder 2.Encouragement and Emotional Support 3.Health Education 4. SM Guidance	22.3%	11.1%	66.7%	77.8%	1	67.2%
W	1.Information Feedback 2.Health Education 3.Disease Data Monitoring 4. SM Guidance	75.0%	66.7%	58.3%	25%	4	25.5%
WD	1.Information Feedback 2.Disease Data Monitoring	55.6%	44.4%	77.8%	88.9%	6	71.6%

HP, Health Provider Involvement, I, Interactivity, P, Personalization, F, Frequency.

According to SUCRA, all E-health interventions resulted in a better control of HbA1c than UC, SMS (SUCRA 67.2%) was ranked as having the highest probability of being the best, followed by SA (SUCRA 37.6%), CM (SUCRA 29.3%), W (SUCRA 25.5%), PC (SUCRA 45.1%) and WD (SUCRA 71.6%) (**Table 2**). Upon fitting a consistency model, the results showed that compared to UC, CM (MD: -0.41, 95% CI: -0.57 to -0.25), PC (MD: -0.32, 95% CI: -0.50 to -0.14), SA (MD: -0.45, 95% CI: -0.61 to -0.30), SMS (MD: - 0.56, 95% CI: -0.82 to -0.31) and W-based interventions (MD: - 0.39, 95% CI: -0.60 to -0.18) helped in reducing patients’ HbA1c levels (%) to a great extent, and the differences were statistically significant (p<0.05). SA (MD: -0.28, 95% CI: -0.55 to -0.01) and SMS (MD: -0.39, 95% CI: -0.73 to -0.04) were more effective in reducing HbA1c than WD (p<0.05) (**Table 3**). **Supplementary Table 9** shows detailed results of both indirect and pairwise comparisons of diabetes management interventions in the network meta-analyses.

**Table 3:** The league table of a network meta-analysis of E-health intervention effects (mean difference MD and 95% confidence intervals).

HbA1c (%)						
**CM**	0.09 (-0.14, 0.33)	-0.04 (-0.26, 0.18)	-0.16 (-0.45, 0.15)	0.02 (-0.24, 0.28)	0.23 (-0.03, 0.5)	0.41 (0.25, 0.57)
-0.09 (-0.33, 0.14)	**PC**	-0.14 (-0.37, 0.09)	-0.25 (-0.54, 0.06)	-0.07 (-0.34, 0.20)	0.14 (-0.15, 0.43)	0.32 (0.14, 0.50)
0.04 (-0.18, 0.26)	0.14 (-0.09, 0.37)	**SA**	-0.11 (-0.41, 0.19)	0.06 (-0.19, 0.31)	0.28 (0.01, 0.55)	0.45 (0.30, 0.61)
0.16 (-0.15, 0.45)	0.25 (-0.06, 0.54)	0.11 (-0.19, 0.41)	**SMS**	0.17 (-0.16, 0.50)	0.39 (0.04, 0.73)	0.56 (0.31, 0.82)
-0.02 (-0.28, 0.24)	0.07 (-0.20, 0.34)	-0.06 (-0.31, 0.19)	-0.17 (-0.50, 0.16)	**W**	0.21 (-0.09, 0.53)	0.39 (0.18, 0.60)
-0.23 (-0.50, 0.03)	-0.14 (-0.43, 0.15)	**-0.28** **(-0.55, -0.01)**	**-0.39** **(-0.73, -0.04)**	-0.21 (-0.53, 0.09)	**WD**	0.18 (-0.05, 0.41)
**-0.41** **(-0.57, -0.25)**	**-0.32** **(-0.50, -0.14)**	**-0.45** **(-0.61, -0.30)**	**-0.56** **(-0.82, -0.31)**	**-0.39** **(-0.60, -0.18)**	-0.18 (-0.41, 0.05)	**UC**

The direct comparison results and indirect comparison results are the intersection of the rows and columns at the location of the interventions. The bolded value in the corresponding columns for WD indicates that the mean differences in HbA1c reductions for SA and SMS compared with WD are statistically significant, respectively.

The bolded value in the corresponding columns for UC indicates that the mean differences in HbA1c reductions for CM, PC, SA, SMS and W compared with UC are statistically significant.

### 3.4 Subgroup analyses

**Figure 4:**
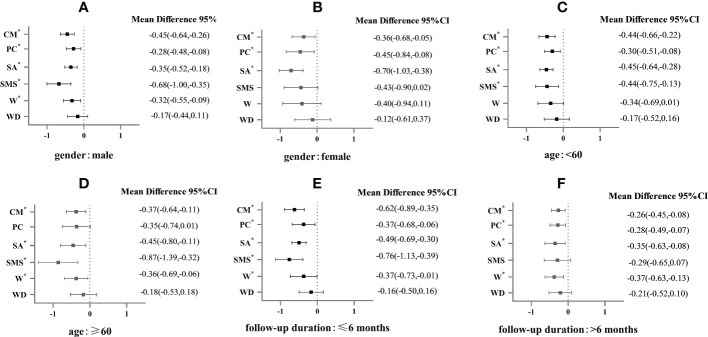
Subgroup analysis of HbA1c (%) in patients with type 2 diabetes in different types of E-health interventions.* mean P<0.05. **(A)** is the E-health intervention effects on the male in the gender subgroup; **(B)** is the E-health intervention effects on the female in the gender subgroup; **(C)** is the E-health intervention effects in patients < 60 years old in the age subgroup; **(D)** is E-health intervention effects in patients ≥ 60 years old in the age subgroup; **(E)** is E-health intervention effects in the subgroup of follow-up time ≤ 6 months; **(F)** is E-health intervention effects in the subgroup of follow-up time > 6 months.

SA was found to be more effective in females (MD -0.70, 95% CI -1.03 to -0.38) than in males (MD -0.35%, 95% CI -0.52 to -0.18), while SMS was found to be more effective in males (MD -0.68, 95% CI -1.00 to -0.35) (**Figures 4A, B**). The results showed that SMS had a better effect on HbA1c reduction in the patients with age ≥ 60 years (MD -0.87, 95% CI -1.39 to -0.32) than the patients under the age of 60 (MD -0.44, 95% CI -0.75 to -0.13) (**Figures 4C, D**). It was found that all interventions, except W and WD, were more effective when the duration was 6 months or shorter (MD -0.76 to -0.37) than when it was longer than 6 months (MD -0.35 to 0.26) (**Figures 4E, F**).

## 4 Discussion

Compared to UC alone, E-health platforms for disease management were more helpful in controlling, SMS was the most effective, followed by SA and CM. In addition, regardless of the form, patients were able to reduce HbA1c levels by 0.18% to 0.56%. E-health has advantages as a tool to guide patients for better disease management, as it provides information regarding glycemic control, dietary and exercise guidance, counseling services, and necessary knowledge on self-management (36). However, compared with traditional methods, such as face-to-face education, E-health has certain prerequisites related to the knowledge level of patient’s and their ability to use technology (37).

### 4.1 SMS and PC-based interventions

SMS and PC are less demanding with regard to the ability of patients to use the technology, PC is more interactive, and SMS is more frequent to convey information; both guide the behavior of patients through information reminders or encouragement, thereby enhancing the efficiency of their actions and motivating individuals (38, 39). One study noted that cueing the behavior of patients using PC had the greatest effect on medication adherence, with patients’ blood glucose levels already under control (at approximately 7%) at 3 months and remaining stable at 9 months (40). Dobler et al. found that the rate of exercise in type 2 diabetes patients increased by 26% through PC and improved their mental health (41).

While PC has highly interactive features, it requires patients to receive interventions at a specific time compared with SMS, which can be sent to patients at any time, can be sent frequently, and are less costly (42). The findings of Nelson et al. are similar to ours; patients preferred SMS to PC, and sending regular SMS can greatly improve their medication adherence (43). High-frequency communication can effectively remind patients to actively adopt SM behaviors, which was confirmed by Peimani et al., who showed that regular messaging achieved better results than personalized message content (44). Another meta-analysis found that SMS interventions helped in reducing the HbA1c levels of patients by 0.38% and that content personalization greatly influenced glycemic control outcomes; however, a high degree of personalization also means a large investment in professionals and technology (45). SMS-based patient behavior management is a cost-effective option that can play a positive role through education and prompting, but the effect of SMS will vary among different behaviors. Middleton and Waller et al. found that SMS alone was insufficient to change the dietary and exercise behaviors of patients but could benefit them in other ways, for example, by improving the frequency of subsequent visits (46, 47).

### 4.2 CM, SA, W, and WD-based interventions

CM, SA, and W provide more integrated services, including monitoring, behavioral guidance, health education, and counseling, which can meet the different needs of patients (48–50). An integrated or combined form of intervention maximizes the ability to bridge the various limitations of E-health interventions and integrate resources, which also means that patients need to acquire sufficient skills to use the technology (51, 52). In contrast, SA is mainly carried out on mobile phones, which is easier for patients to accept (49). Wang et al. showed that after the SA intervention, patients had a significant increase in knowledge and a reduction in HbA1c levels from 8.62% to 7.12%, as well as a decrease in rehospitalization and medical expenditures (53). Hilmarsdóttir et al. found the blood glucose levels and symptoms such as anxiety and depression were effectively reduced in patients, but the app usage of patients gradually decreased as their blood glucose levels stabilized (54). Bowls et al. found that the participation of patients in app usage was difficult to guarantee, with only 9.9%-17.0% of patients participating; owing to a lack of personalized guidance and forgetfulness, HbA1c levels of the intervention group were only reduced by 0.10% after 6 months (55).

The content provided by the web-based interventions is similar to the use of SA. Vaughan et al. found that the HbA1c levels of 88.57% patients in the intervention group reduced by more than 0.50% through intervention (56). However, the website presents similar problems as SA; patients are reluctant to use them or discontinue use before the end of the study due to objective barriers, such as not being familiar with the use of phones, complexity, and a lack of health literacy (57). WD, such as flash glucose monitoring, is mainly used for continuous blood glucose and blood pressure monitoring. Patients adjust their SM behavior in response to the changes in blood glucose levels, which prevents adverse consequences caused by hypoglycemia (58). In addition, this approach is subject to high technical requirements, and adverse events associated with the device, such as redness and itching, can reduce patient use and the effect of intervention on glycemic control (59).

### 4.3 Subgroup analysis

Using subgroup analysis, we discovered that females benefitted more from glycemic control through E-health interventions in general, and younger patients (age <60) benefitted more from complex interventions such as CM and SA. Previous studies have found that women are more concerned about their health and more willing to seek health information than are men; similarly, younger patients are more receptive to new things and technology, face fewer obstacles to technology and are more likely to learn and use e-technology for disease management (60).

Furthermore, the effect was stronger at an intervention duration of under 6 months than at that longer than 6 months, which was seen in several studies (61, 62). A study using SMS to provide patient interventions showed that patients had control of HbA1c in the short term, with a reduction of -0.24%; after 6 months (63). The effectiveness of disease management depends mainly on the extent to which patients participate; after using services for a while there is a decrease in motivation and willingness, which affects the subsequent outcome (64).

### 4.4 Strengths and limitations

This study explored the differences in the effects of different forms of E-health interventions on glycemic control by direct and indirect comparison through Bayesian network meta-analysis. Compared to the frequency-based method in parameter estimation, which has the drawback of instability due to constant iterative estimation of the maximum likelihood function, the Bayesian method can reduce the risk of biased results and is more flexible (65). This study had a large sample size, analyzed the characteristics of several types of E-health, and summarized their personalization, interactivity, content, and frequency.

Some limitations of this study must be acknowledged. First, the RCTs included in this study were at high risk for blinding and group concealment; however, considering that communication between participants and personnel is part of many E-health interventions, bias around blinding may be inevitable compared with that in studies evaluating drug treatment effects. Second, funnel plots are not perfectly symmetrical and may exhibit publication bias. However, the trim and fill method and Egger test for each E-health group revealed that the model was robust and that this factor did not affect the results of the study. The asymmetry of funnel plots may be due to the low quality, small sample size, and heterogeneity of the included studies (66). Third, heterogeneity was found in this study (I^2^ = 76.0%). After meta-regression and subgroup analyses, gender (I^2^ = 49.7%) and race (I^2^ = 41.6%) could explain the source of heterogeneity. Classification of more than two interventions as CM may also be a reason for the high heterogeneity (67). Finally, although this study defined the intervention content, the frequency of intervention, health provider involvement, personalization, and interactivity were not included in the network analysis. Concurrently, because most of the included studies targeted interventions for comprehensive SM behaviors, this study did not explore the role of E-health in different behaviors. More research is needed in the future to explore the effects of different characteristics of e-health on glycemic control.

## 5 Conclusion

This study comprehensively evaluated the effects of six forms of E-health interventions in patients with type 2 diabetes. All forms have certain advantages in disease management; these include high service efficiency and more convenient provision of health information. However, they differ in service content, frequency, and persistence of effects. Future research should fully investigate the advantages of various forms and combinations of intervention methods to maximize the effectiveness of disease management to ensure scientific and professional health. While focusing on the extent of the impact of E-health, it is also essential to seek long-term management mechanisms that are sustainable.

## Data availability statement

The raw data supporting the conclusions of this article will be made available by the authors, without undue reservation.

## Author contributions

XZ and LZ completed data extraction, data analysis, and thesis writing. YHL, YXL, and XCY participated in the mitigation of data extraction and analysis, helped extract specific content, and assisted in writing. WC assisted in the design of the study and assisted in the systematic review of the manuscript. YJ helped interpret the findings and reviewed and edited the manuscript. CC helped with the study design, assisted in the interpretation of the study results, and reviewed the whole process. Everyone has full access to all data in the study and is responsible for the integrity of the data and the accuracy of the data analysis. All authors contributed to the article and approved the submitted version.
