# Supplementary material for: Effects of E-health-based interventions on glycemic control for patients with type 2 diabetes: a Bayesian network meta-analysis
Source: Front Endocrinol (Lausanne). 2023 May 4;14:1068254. doi: 10.3389/fendo.2023.1068254 (PMC10196691; doi:10.3389/fendo.2023.1068254)
Supplement: Supplementary file 1 [file DataSheet_1.doc]

**Supplementary Material**

**Supplementary Table1:** Search Strategy

| Pubmed | (((((Type 2 diabetes mellitus[tiab]) OR (Type 2[tiab]) OR (Type 2 diabetes[tiab]) OR (T2DM[tiab])) AND ((mobile application[tiab]) OR (mobile applications[tiab]) OR (mobile app[tiab]) OR (mobile apps[tiab]) OR (portable[tiab]) OR (portable software[tiab]) OR (portable app[tiab]) OR (portable software[tiab]) OR (portable application[tiab]) OR (portable software application[tiab]) OR (portable electronic app[tiab]) OR (portable electronic[tiab]) OR (application[tiab]) OR (app[tiab]) OR (apps[tiab]) OR (applications[tiab]) OR (software app[tiab]) OR (software[tiab]) OR (software application[tiab]) OR (smartphone app[tiab]) OR (smartphone apps) OR (electronic[tiab]) OR (electronic app[tiab]) OR (e-health[tiab]) OR (online[tiab]) OR (phone[tiab]) OR (mobile phone[tiab]) OR (cell phone[tiab]) OR (digital[tiab]) OR (digital behavior change[tiab]) OR (digital health[tiab]) OR (telehealth[tiab]) OR (telemedicine[tiab]) OR (smartphone[tiab]) OR (internet intervention[tiab]) OR (electronic health[tiab]) OR (wearable technology[tiab]))) AND ((glucose[tiab]) OR (blood glucose[tiab]) OR (glycaemia[tiab]) OR (glycemic[tiab]) OR (glycaemic[tiab]) OR (HbA1c[tiab]) OR (A1c[tiab]) OR (glycated[tiab]))) AND ((randomized controlled trial[tiab]) OR (controlled clinical trial[tiab]))) |
| --- | --- |
| Embase |  |
| #1 | ‘Type 2 diabetes mellitus’:ab,ti OR ‘Type 2’:ab,ti OR ‘Type 2 diabetes’:ab,ti OR ‘T2DM’:ab,ti |
| #2 | 'mobile application':ab,ti OR 'mobile applications':ab,ti OR 'mobile app':ab,ti OR 'mobile apps':ab,ti OR 'portable':ab,ti OR 'portable app':ab,ti OR 'portable software':ab,ti OR 'portable application':ab,ti OR 'portable software application':ab,ti OR 'portable electronic app':ab,ti OR 'portable electronic':ab,ti OR 'application':ab,ti OR 'app':ab,ti OR 'apps':ab,ti OR 'applications':ab,ti OR 'software app':ab,ti OR 'software':ab,ti OR 'software application':ab,ti OR 'smartphone app':ab,ti OR 'smartphone apps':ab,ti OR 'electronic':ab,ti OR 'electronic app':ab,ti OR 'e-health':ab,ti OR 'online':ab,ti OR 'phone':ab,ti OR 'mobile phone':ab,ti OR 'cell phone':ab,ti OR 'digital':ab,ti OR 'digital behavior change':ab,ti OR 'digital health':ab,ti OR 'telehealth':ab,ti OR 'telemedicine':ab,ti OR 'smartphone':ab,ti OR 'internet intervention':ab,ti OR 'electronic health':ab,ti OR 'wearable technology':ab,ti |
| #3 | 'glucose':ab,ti OR 'blood glucose':ab,ti OR 'glycaemia':ab,ti OR 'glycemic':ab,ti OR 'glycaemic':ab,ti OR 'hba1c':ab,ti OR 'a1c':ab,ti OR 'glycated':ab,ti |
| #4 | 'randomized controlled trial':ab,ti OR 'controlled clinical trial':ab,ti |
| #5 | #1 AND #2 AND #3 AND #4 |
| Cochrane |  |
| #1 | (Diabetes Mellitus, Type 2):ti,ab,kw OR (Type 2):ti,ab,kw OR (Type 2 diabetes):ti,ab,kw OR (T2DM):ti,ab,kw |
| #2 | (mobile application):ab,ti,kw OR (mobile applications):ab,ti,kw OR (mobile app):ab,ti,kw OR (mobile apps):ab,ti,kw OR (portable):ab,ti,kw OR (portable software):ab,ti,kw OR (portable app):ab,ti,kw OR (portable software):ab,ti,kw OR (portable application):ab,ti,kw OR (portable software application):ab,ti,kw OR (portable electronic app):ab,ti,kw OR (portable electronic):ab,ti,kw OR (application):ab,ti,kw OR (app):ab,ti,kw OR (apps):ab,ti,kw OR (applications):ab,ti,kw OR (software app):ab,ti,kw OR (software):ab,ti,kw OR (software application):ab,ti,kw OR (smartphone app):ab,ti,kw OR (smartphone apps):ab,ti,kw OR (electronic):ab,ti,kw OR (electronic app):ab,ti,kw OR (e-health):ab,ti,kw OR (online):ab,ti,kw OR (phone):ab,ti,kw OR (mobile phone):ab,ti,kw OR (cell phone):ab,ti,kw OR (digital):ab,ti,kw OR (digital behavior change):ab,ti,kw OR (digital health):ab,ti,kw OR (telehealth):ab,ti,kw OR (telemedicine):ab,ti,kw OR (smartphone):ab,ti,kw OR (internet intervention):ab,ti,kw OR (electronic health):ab,ti,kw OR (wearable technology):ab,ti,kw |
| #3 | (glucose):ab,ti,kw OR (blood glucose):ab,ti,kw OR (glycaemia):ab,ti,kw OR (glycemic):ab,ti,kw OR (glycaemic):ab,ti,kw OR (HbAlc):ab,ti,kw OR (ALc):ab,ti,kw OR (glycated):ab,ti,kw |
| #4 | #1 AND #2 AND #3 |

**Supplementary Table2: The characteristics of the studies**

| **Country** | **Study** | **Sample**  **Size** | **Treatment** | **Time** | **Age** | **Gender** | **Race** |
| --- | --- | --- | --- | --- | --- | --- | --- |
| Spain | Agboola2016 | 64 | CM | 6 | 51.4 | 48.4% | White |
|  | Agboola2016 | 62 | EM |  |  |  |  |
| Oman | Alghafri2018 | 82 | CM | 12 | 44.2 | 40.9% | White |
|  | Alghafri2018 | 92 | UC |  |  |  |  |
| Ghana | Asante2020 | 30 | PC | 3 | 55.8 | 21.7% | Black |
|  | Asante2020 | 30 | UC |  |  |  |  |
| Bulgaria | Brodnik2014 | 53 | CM | 12 | 55.5 | 60.8% | White |
|  | Brodnik2014 | 54 | UC |  |  |  |  |
| UK | Bentley2016 | 8 | EM | 4 | 52.9 | 44.4% | White |
|  | Bentley2016 | 5 | CM |  |  |  |  |
|  | Bentley2016 | 7 | UC |  |  |  |  |
| Netherlands | Boels2019 | 114 | SA | 6 | 59.2 | 60.5% | White |
|  | Boels2019 | 115 | UC |  |  |  |  |
| Poland | Bujnowska-Fedak2010 | 47 | EM | 6 | 55.3 | 53.7% | White |
|  | Bujnowska-Fedak2010 | 48 | UC |  |  |  |  |
| China | Carallo2017 | 93 | W | 6 | 53.7 | 62.5% | Asian |
|  | Carallo2017 | 85 | UC |  |  |  |  |
| Korea | CHO2006 | 35 | W | 30 | 52.9 | 61.3% | Asian |
|  | CHO2006 | 36 | UC |  |  |  |  |
| Korea | Cho2011 | 36 | CM | 3 | 64.2 | 39.0% | Asian |
|  | Cho2011 | 35 | UC |  |  |  |  |
| America | Crowley2016 | 23 | PC | 6 | 60.0 | 96.0% | Black |
|  | Crowley2016 | 23 | UC |  |  |  |  |
| Germany | Döbler2018 | 98 | PC | 12 | 51.9 | 70.4% | White |
|  | Döbler2018 | 101 | UC |  |  |  |  |
| Italy | Dario2017 | 168 | W | 12 | 73.0 | 73.4% | White |
|  | Dario2017 | 78 | UC |  |  |  |  |
| America | Elizabeth2020 | 41 | SA | 6 | 54.9 | 28.1% | White |
|  | Elizabeth2020 | 42 | UC |  |  |  |  |
| China | Fang2017 | 51 | SMS | 12 | 57.3 | 82.2% | Asian |
|  | Fang2017 | 58 | PC |  |  |  |  |
| UK | Farmer2021 | 510 | SMS | 12 | 57.1 | 30.1% | Black |
|  | Farmer2021 | 511 | UC |  |  |  |  |
| Spain | Fortmann2017 | 50 | SMS | 6 | 48.5 | 25.4% | White |
|  | Fortmann2017 | 59 | UC |  |  |  |  |
| Australia | Furler2020 | 130 | EM | 12 | 60.1 | 58.5% | White |
|  | Furler2020 | 137 | UC |  |  |  |  |
| America | GLASGOW2000 | 67 | PC | 6 | 59.9 | 43.2% | Asian |
|  | GLASGOW2000 | 67 | UC |  |  |  |  |
| Australia | Gong2020 | 85 | SA | 12 | 56.9 | 58.3% | White |
|  | Gong2020 | 87 | UC |  |  |  |  |
| Iran | Goodarzi2012 | 43 | SMS | 3 | 53.7 | 22.2% | White |
|  | Goodarzi2012 | 38 | UC |  |  |  |  |
| China | Guo2021 | 32 | CM | 1 | 57.4 | 60.9% | Asian |
|  | Guo2021 | 32 | UC |  |  |  |  |
| Germany | Haak2017 | 139 | EM | 6 | 59.2 | 66.7% | White |
|  | Haak2017 | 62 | UC |  |  |  |  |
| America | Heisler2014 | 86 | W | 3 | 51.5 | 28.7% | White |
|  | Heisler2014 | 89 | UC |  |  |  |  |
| Iceland | Hilmarsdóttir2021 | 15 | SA | 6 | 51.2 | 40.0% | White |
|  | Hilmarsdóttir2021 | 15 | UC |  |  |  |  |
| Norway | Holmen2014 | 39 | SA | 12 | 57.3 | 63.4% | White |
|  | Holmen2014 | 41 | UC |  |  |  |  |
| China | Hu2021 | 72 | SA | 6 | 51.1 | 66.2% | Asian |
|  | Hu2021 | 70 | UC |  |  |  |  |
| Singapore | Huang2019 | 19 | SA | 3 | 51.7 | 48.8% | Asian |
|  | Huang2019 | 15 | UC |  |  |  |  |
| America | Jing Wang2018 | 10 | SA | 6 | 55.4 | 27.8% | White |
|  | Jing Wang2018 | 6 | UC |  |  |  |  |
| Turkey | Kasar2022 | 31 | CM | 3 | 54.0 | 41.9% | White |
|  | Kasar2022 | 32 | UC |  |  |  |  |
| Germany | Kempf2017 | 93 | PC | 13 | 59.4 | 53.9% | White |
|  | Kempf2017 | 74 | UC |  |  |  |  |
| America | Kerfoot2017 | 227 | SA | 12 | 59.5 | 93.9% | White |
|  | Kerfoot2017 | 229 | UC |  |  |  |  |
| Korea | Kim2007 | 25 | CM | 3 | 47.2 | 43.1% | Asian |
|  | Kim2007 | 26 | UC |  |  |  |  |
| Korea | Kim2019 | 40 | SA | 3 | 51.3 | 47.1% | Asian |
|  | Kim2019 | 37 | PC |  |  |  |  |
|  | Kim2019 | 43 | UC |  |  |  |  |
| Netherlands | Kooiman2018 | 36 | EM | 3 | 56.4 | 52.8% | White |
|  | Kooiman2018 | 30 | UC |  |  |  |  |
| Korea | Ku2020 | 17 | SA | 3 | 49.9 | 35.0% | Asian |
|  | Ku2020 | 18 | UC |  |  |  |  |
| India | Kumar2021 | 150 | SA | 6 | 64.7 | 60.0% | Muti-racial |
|  | Kumar2021 | 150 | UC |  |  |  |  |
| Indonesia | Kusnanto 2019 | 15 | SA | 3 | 50.0 | 43.3% | Asian |
|  | Kusnanto 2019 | 15 | UC |  |  |  |  |
| Korea | Lee2018 | 72 | CM | 6 | 52.0 | 63.2% | Asian |
|  | Lee2018 | 64 | UC |  |  |  |  |
| Malaysia | Lee2019 | 104 | CM | 13 | 56.2 | 45.0% | Asian |
|  | Lee2019 | 104 | UC |  |  |  |  |
| China | Li2021 | 41 | CM | 3 | 48.2 | 76.2% | Asian |
|  | Li2021 | 41 | UC |  |  |  |  |
| Korea | Lim2016 | 43 | CM | 6 | 65.1 | 75.0% | Asian |
|  | Lim2016 | 42 | UC |  |  |  |  |
| Singapore | Lim2021 | 105 | SA | 6 | 51.2 | 64.7% | Asian |
|  | Lim2021 | 99 | UC |  |  |  |  |
| China | Lin2021 | 58 | CM | 6 | 62.0 | 50.9% | Asian |
|  | Lin2021 | 56 | UC |  |  |  |  |
| Sweden | Lindberg2017 | 50 | W | 19 | 67.5 | 70.4% | White |
|  | Lindberg2017 | 72 | UC |  |  |  |  |
| China | Lyu2021 | 54 | W | 3 | 60.9 | 48.1% | Asian |
|  | Lyu2021 | 52 | UC |  |  |  |  |
| Spain | Marı´a 2009 | 146 | CM | 12 | 63.9 | 52.0% | White |
|  | Marı´a 2009 | 151 | UC |  |  |  |  |
| America | McMahon2012 | 51 | SA | 12 | 60.2 | 94.7% | White |
|  | McMahon2012 | 51 | PC |  |  |  |  |
|  | McMahon2012 | 49 | W |  |  |  |  |
| Australia | Middleton2021 | 16 | SMS | 12 | 32.7 | 50.0% | White |
|  | Middleton2021 | 11 | UC |  |  |  |  |
| America | Millan-Ferro2020 | 107 | PC | 6 | 55.2 | 44.9% | White |
|  | Millan-Ferro2020 | 116 | UC |  |  |  |  |
| Italy | Nicolucci2015 | 114 | CM | 12 | 58.4 | 61.5% | White |
|  | Nicolucci2015 | 135 | UC |  |  |  |  |
| Belgium | Odnoletkova2016 | 240 | PC | 18 | 63.1 | 61.5% | White |
|  | Odnoletkova2016 | 246 | UC |  |  |  |  |
| Finland | Orsama2013 | 24 | CM | 10 | 61.9 | 54.2% | White |
|  | Orsama2013 | 24 | UC |  |  |  |  |
| India | Patnaik2021 | 33 | SA | 24 | 42.3 | 65.2% | Muti-racial |
|  | Patnaik2021 | 33 | W |  |  |  |  |
| Thailand | Poonprapai2022 | 78 | SA | 9 | 67.6 | 40.1% | Asian |
|  | Poonprapai2022 | 79 | UC |  |  |  |  |
| America | Presley2020 | 62 | SA | 6 | 55.5 | 28.9% | Black |
|  | Presley2020 | 35 | UC |  |  |  |  |
| Malaysia | Ramadas2018 | 63 | W | 6 | 50.5 | 68.5% | Asian |
|  | Ramadas2018 | 55 | UC |  |  |  |  |
| America | Rosas2017 | 87 | CM | 9 | 54.2 | 36.5% | White |
|  | Rosas2017 | 88 | UC |  |  |  |  |
| Iran | Sarayani2018 | 40 | PC | 9 | 55.1 | 58.6% | White |
|  | Sarayani2018 | 44 | UC |  |  |  |  |
| Iran | Shahsavari2020 | 30 | PC | 3 | 62.6 | 28.3% | White |
|  | Shahsavari2020 | 30 | UC |  |  |  |  |
| Canada | Sherifali2020 | 186 | PC | 12 | 57.9 | 49.9% | White |
|  | Sherifali2020 | 171 | UC |  |  |  |  |
| Latvia | Sokolovska2020 | 14 | CM | 4 | 60.9 | 32.5% | White |
|  | Sokolovska2020 | 26 | UC |  |  |  |  |
| UK | Steventon2014 | 266 | EM | 12 | 64.9 | 57.9% | White |
|  | Steventon2014 | 191 | UC |  |  |  |  |
| America | STONE2009 | 64 | CM | 6 | 55.0 | 98.5% | White |
|  | STONE2009 | 73 | PC |  |  |  |  |
| Germany | Storch,2019 | 52 | PC | 12 | 58.9 | 81.7% | White |
|  | Storch,2019 | 54 | UC |  |  |  |  |
| China | Sun2019 | 44 | SA | 6 | 68.0 | 40.4% | Asian |
|  | Sun2019 | 47 | UC |  |  |  |  |
| Canada | Tildesley2011 | 23 | W | 12 | 59.5 | 63.0% | White |
|  | Tildesley2011 | 23 | UC |  |  |  |  |
| Turkey | Timurtas2022 | 25 | SA | 3 | 51.5 | NA | White |
|  | Timurtas2022 | 24 | EM |  |  |  |  |
| America | Trief2016 | 69 | PC | 12 | 56.8 | 61.6% | Muti-racial |
|  | Trief2016 | 57 | UC |  |  |  |  |
| French | Turnin2021 | 128 | W | 12 | 59.5 | 63.1% | White |
|  | Turnin2021 | 135 | UC |  |  |  |  |
| India | Vinitha2019 | 126 | SMS | 24 | 43.2 | 67.7% | White |
|  | Vinitha2019 | 122 | UC |  |  |  |  |
| America | Wakefield2014 | 41 | EM | 6 | 60.0 | 44.4% | White |
|  | Wakefield2014 | 53 | UC |  |  |  |  |
| Japan | Waki2014 | 27 | SA | 3 | 57.3 | 75.9% | Asian |
|  | Waki2014 | 27 | UC |  |  |  |  |
| Australia | Waller2021 | 186 | SMS | 6 | 62.0 | 51.2% | White |
|  | Waller2021 | 189 | UC |  |  |  |  |
| China | Wang2019 | 60 | SA | 6 | 45.4 | 53.3% | Asian |
|  | Wang2019 | 60 | UC |  |  |  |  |
| Singapore | Wang2020 | 20 | SA | 3 | 52.5 | 51.0% | Asian |
|  | Wang2020 | 20 | UC |  |  |  |  |
| UK | Wild2016 | 146 | CM | 9 | 61.0 | 66.7% | White |
|  | Wild2016 | 139 | UC |  |  |  |  |
| Australia | Williams2012 | 51 | PC | 6 | 57.4 | 62.5% | White |
|  | Williams2012 | 55 | UC |  |  |  |  |
| China | Xia2022 | 64 | SMS | 6 | 60.0 | 63.3% | Asian |
|  | Xia2022 | 56 | UC |  |  |  |  |
| America | Xu2020 | 19 | SMS | 6 | 55.0 | 31.4% | White |
|  | Xu2020 | 18 | UC |  |  |  |  |
| Korea | Yang2020 | 145 | SA | 3 | 56.7 | 50.6% | Asian |
|  | Yang2020 | 94 | UC |  |  |  |  |
| China | Yang2021 | 47 | W | 12 | 66.2 | 40.2% | Asian |
|  | Yang2021 | 50 | UC |  |  |  |  |
| Korea | Yeojin2022 | 32 | SA | 2 | 55.2 | 44.1% | Asian |
|  | Yeojin2022 | 36 | UC |  |  |  |  |
| Spain | Yolanda2020 | 375 | CM | 24 | 55.5 | 50.6% | White |
|  | Yolanda2020 | 471 | UC |  |  |  |  |
| America | Young2017 | 141 | EM | 12 | 61.0 | 46.0% | White |
|  | Young2017 | 139 | CM |  |  |  |  |
|  | Young2017 | 147 | UC |  |  |  |  |
| China | Yu2019 | 48 | SA | 6 | 54.1 | 69.9% | Asian |
|  | Yu2019 | 47 | UC |  |  |  |  |
| China | Zhai2020 | 60 | SA | 6 | 54.9 | 52.2% | Asian |
|  | Zhai2020 | 58 | UC |  |  |  |  |
| China | Zhou2014 | 53 | W | 3 | NA | NA | Asian |
|  | Zhou2014 | 55 | UC |  |  |  |  |

Note: Single race refers to the main race of baseline population, which can be judged from the baseline data of the article (if it included ethnic composition) or the main ethnic groups in the study area (if it did not include ethnic composition)

Muti-racial means that the article explicitly referred to multi ethnic groups, but did not provide baseline data including ethnic composition.

**
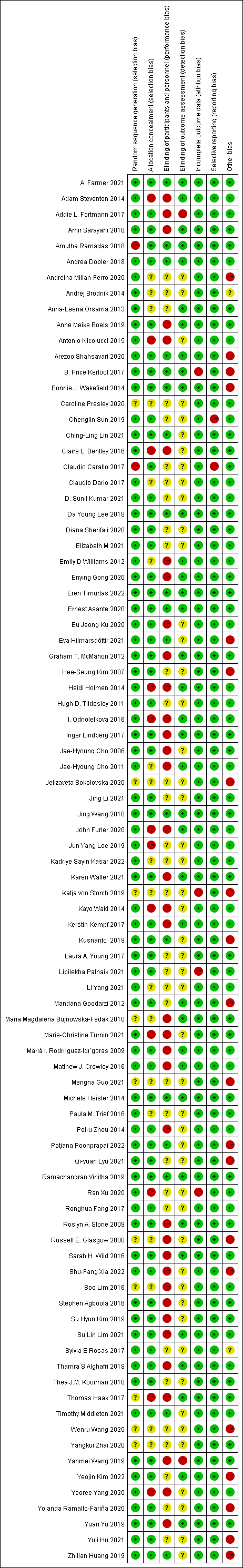
**

**Supplementary Figure 1:** Risk of bias of individual trials


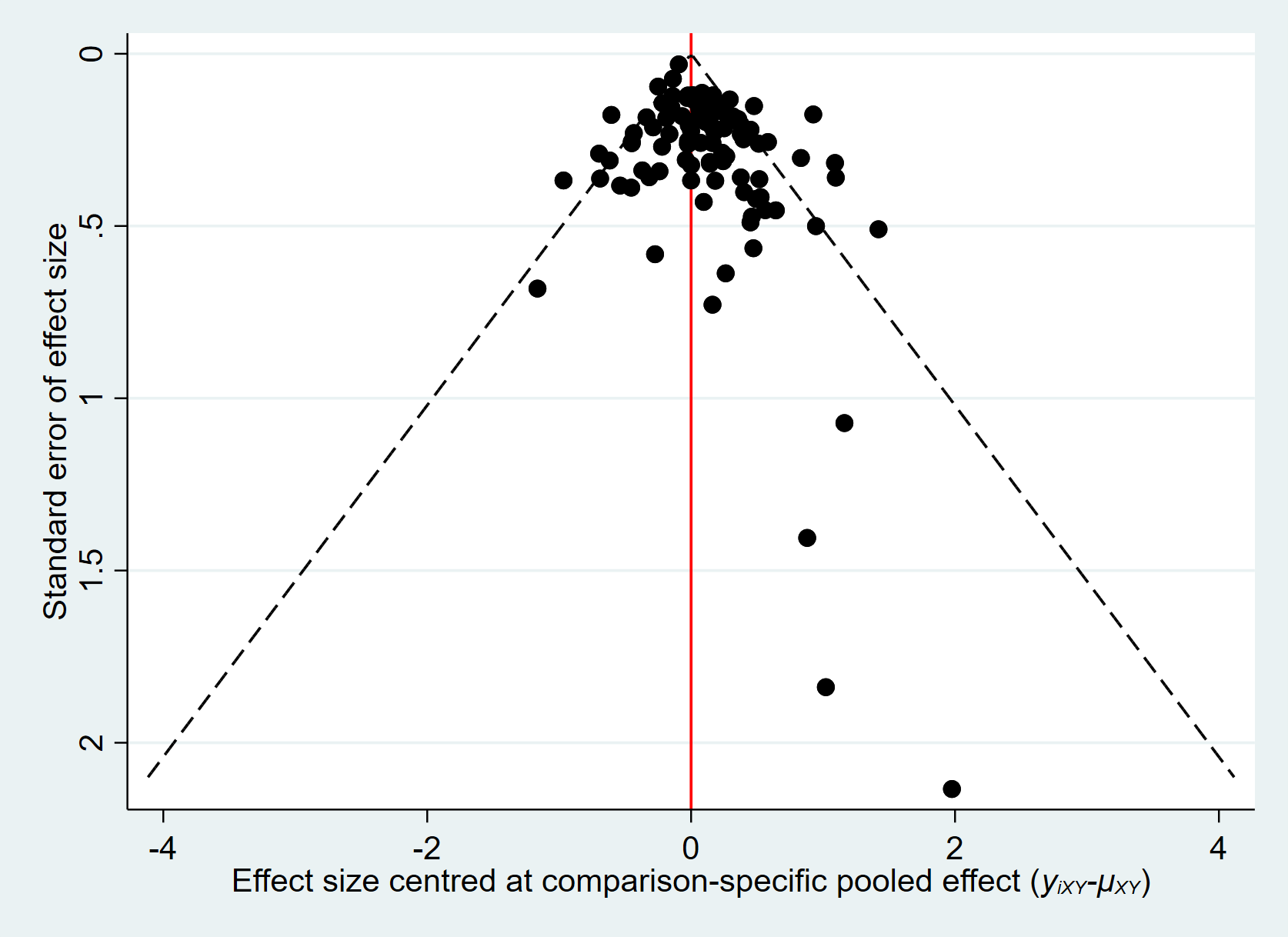
**Supplementary Figure 2** Comparison-adjusted funnel plot for HbA1c involving all studies comparing all E-health interventions vs. usual care.

**Supplementary Table3** Egger test for Comprehensive Measures vs. Usual Care.

| Number of studies=22 | |  |  | Root MSE =1.569 | |
| --- | --- | --- | --- | --- | --- |
| Std_Eff | Coef. | Std. Err. | t | P | 95% CI |
| **slope** | **0.05** | **0.13** | **0.37** | **0.714** | **(-0.22,0.31)** |
| bias | -1.58 | 0.80 | -1.97 | **0.062** | (-3.24,0.09) |


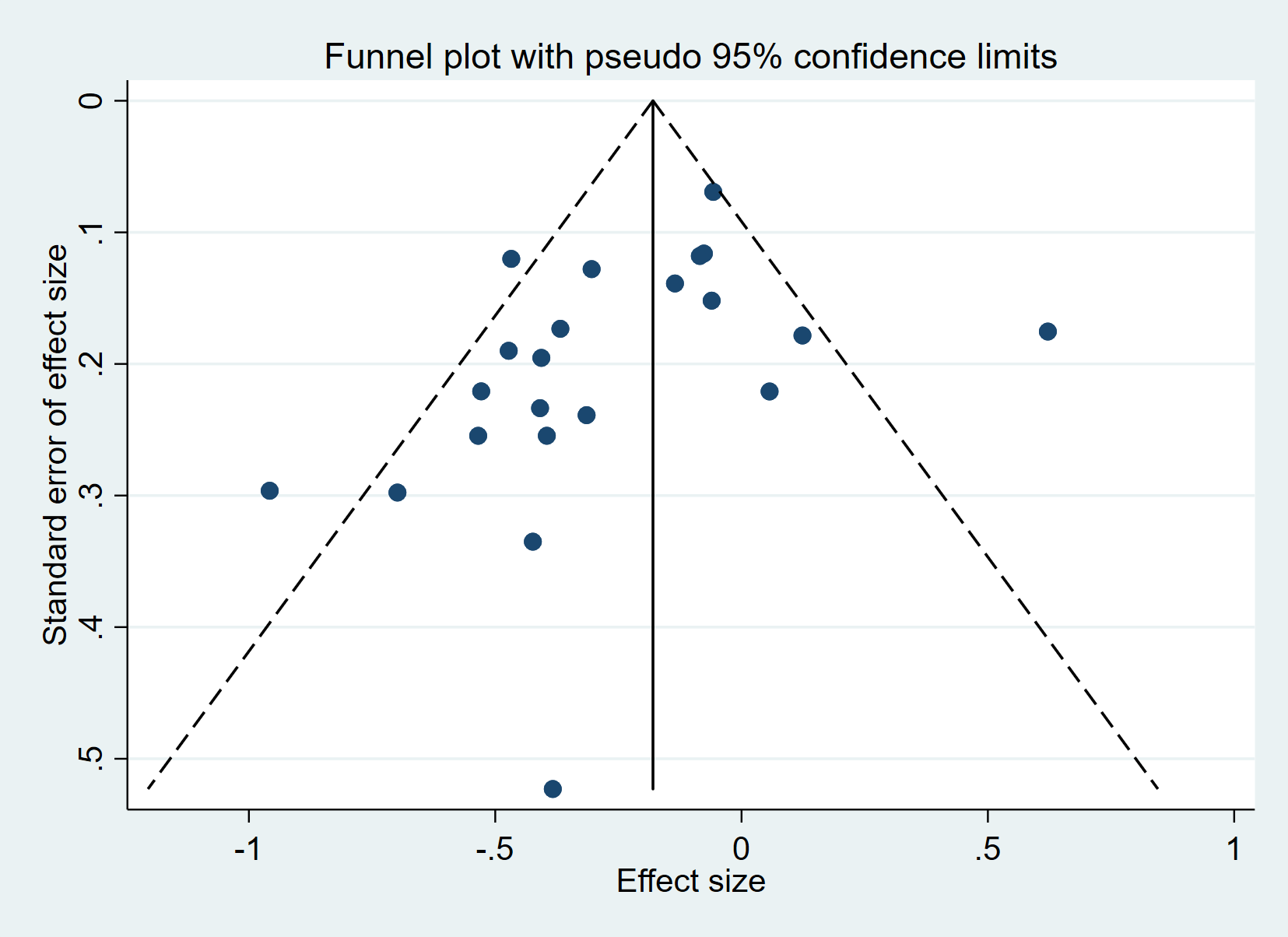
**Supplementary Figure 3** Comparison-adjusted funnel plot for Comprehensive Measures vs. Usual Care.

**Supplementary Table 4** Egger test for Phone Call vs. Usual Care.

| Number of studies=17 | |  |  | Root MSE =1.874 | |
| --- | --- | --- | --- | --- | --- |
| **Std_Eff** | **Coef.** | **Std. Err.** | **t** | **P** | **95% CI** |
| slope | 0.06 | 0.23 | 0.27 | 0.788 | (-0.43,0.56) |
| bias | -2.20 | 1.41 | -1.56 | **0.139** | (-5.21,0.80) |


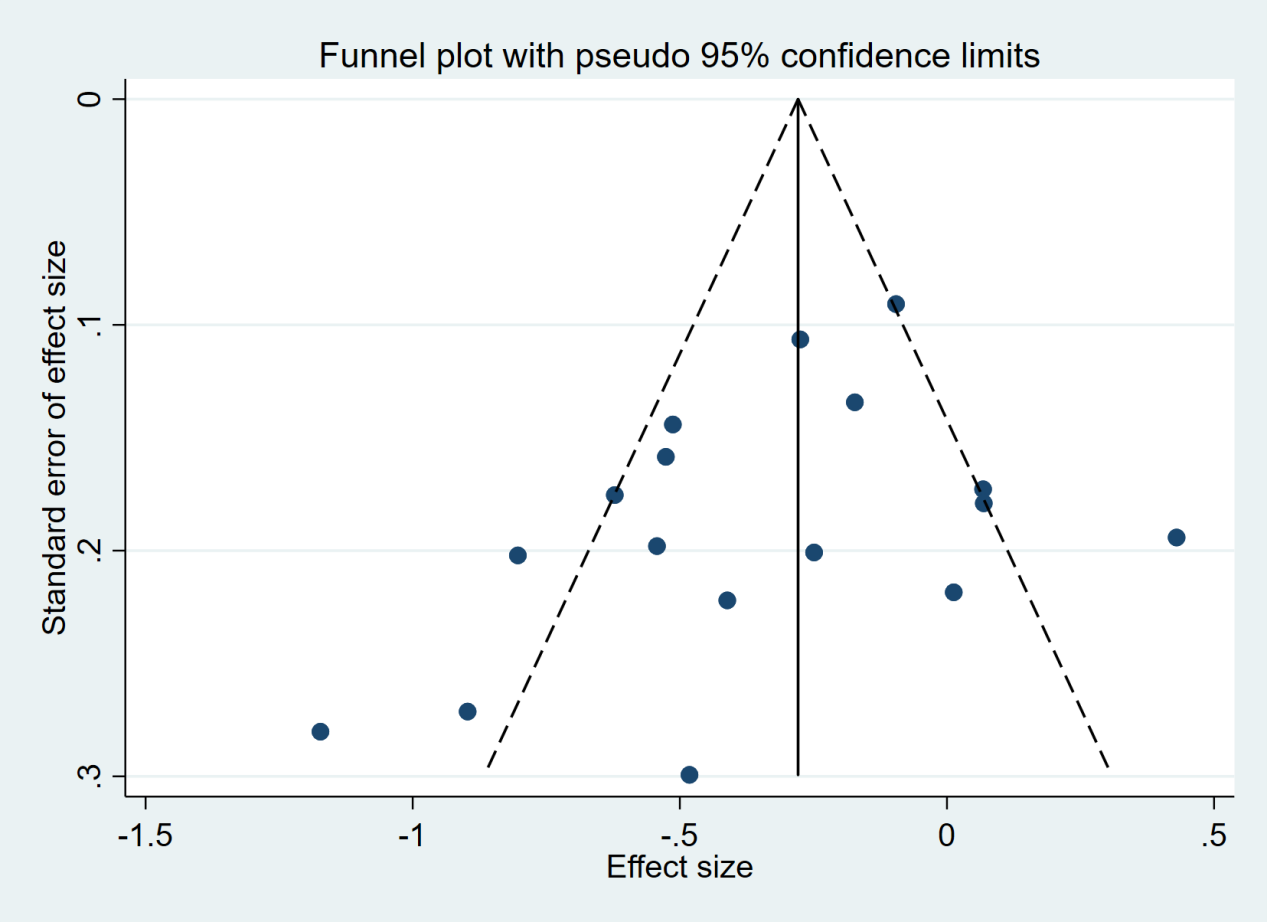
**Supplementary Figure 4** Comparison-adjusted funnel plot for Phone Call vs. Usual Care.

**Supplementary Table 5** Egger test for Smartphone Application vs. Usual Care.

| Number of studies=27 | |  |  | Root MSE =1.309 | |
| --- | --- | --- | --- | --- | --- |
| **Std_Eff** | **Coef.** | **Std. Err.** | **t** | **P** | **95% CI** |
| slope | -0.12 | 0.13 | -0.97 | 0.343 | (-0.38,0.14) |
| bias | -0.87 | 0.68 | -1.28 | **0.213** | (-2.26,0.53) |


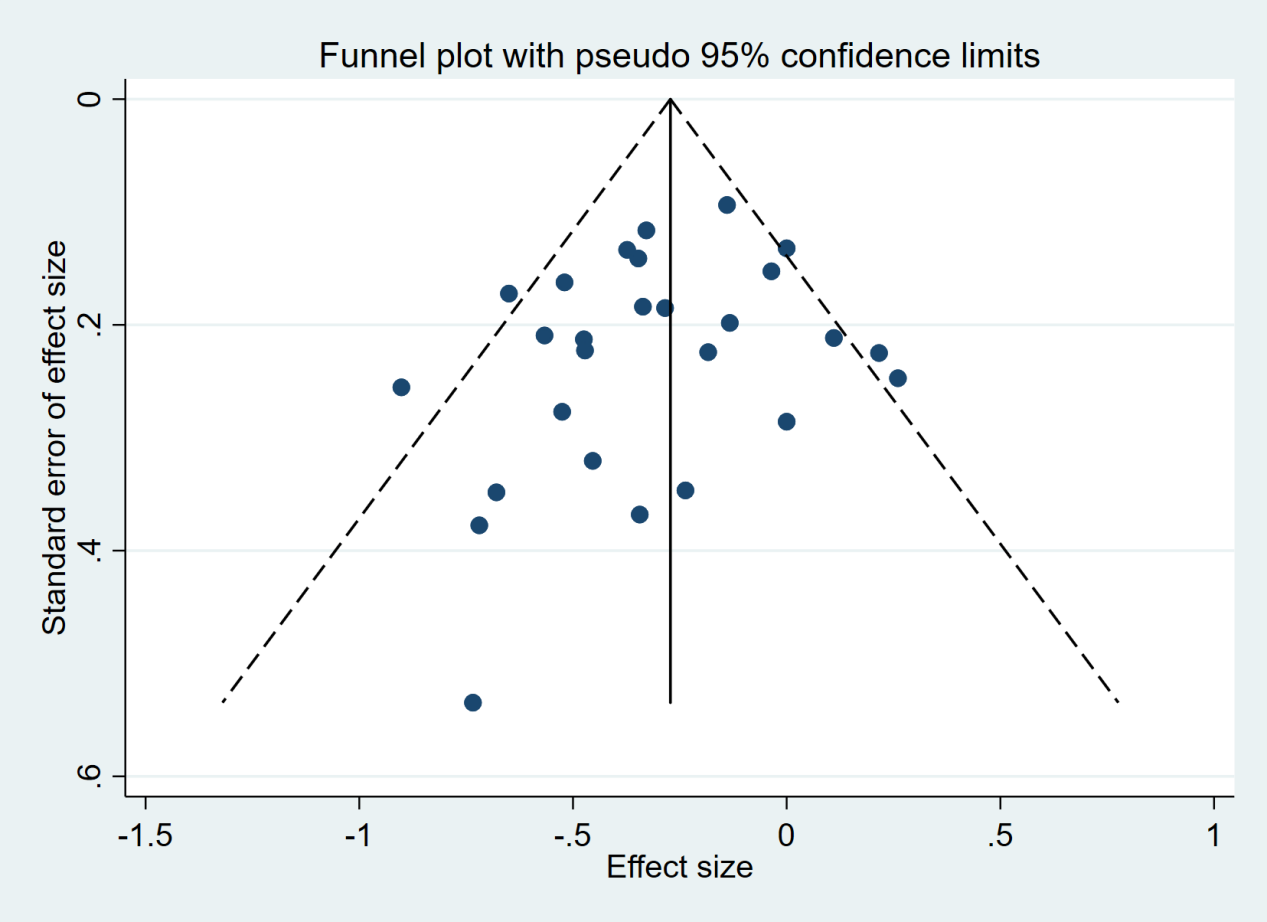
**Supplementary Figure 5** Comparison-adjusted funnel plot for Smartphone Application vs. Usual Care.

**Supplementary Table 6** Egger test for Short Message Services vs. Usual Care.

| Number of studies=9 | |  |  | Root MSE =2.364 | |
| --- | --- | --- | --- | --- | --- |
| **Std_Eff** | **Coef.** | **Std. Err.** | **t** | **P** | **95% CI** |
| slope | 0.16 | 0.20 | 0.80 | 0.448 | (-0.31,0.63) |
| bias | -2.90 | 1.52 | -1.91 | **0.098** | (-6.48,0.69) |


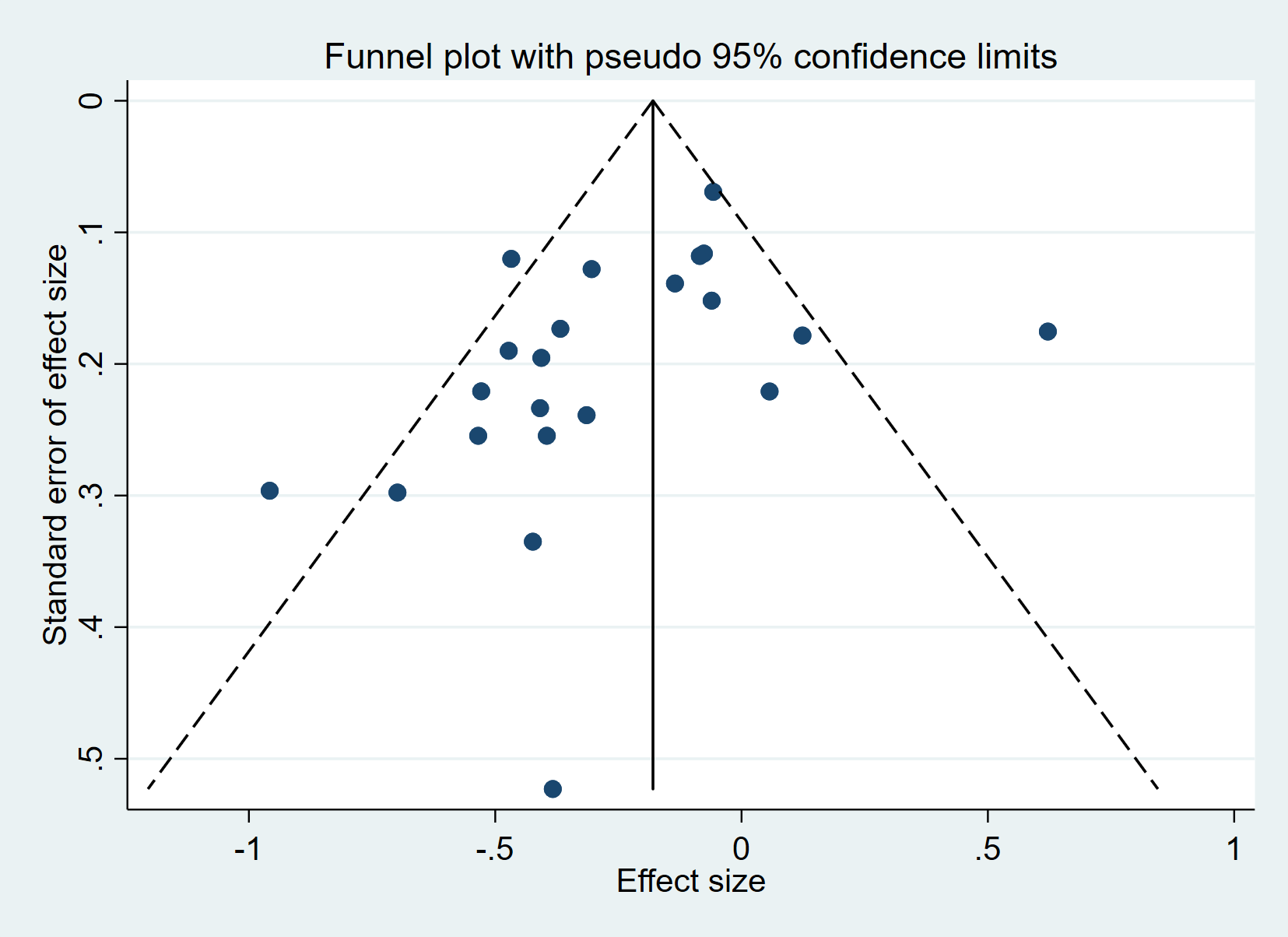
**Supplementary Figure 6** Comparison-adjusted funnel plot for Short Message Services vs. Usual Care.

**Supplementary Table 7** Egger test for Websites vs. Usual Care.

| Number of studies=13 | |  |  | Root MSE =1.311 | |
| --- | --- | --- | --- | --- | --- |
| **Std_Eff** | **Coef.** | **Std. Err.** | **t** | **P** | **95% CI** |
| slope | 0.16 | 0.28 | 0.58 | 0.571 | (-0.45,0.78) |
| bias | -2.43 | 1.56 | -1.56 | **0.148** | (-5.87,1.01) |


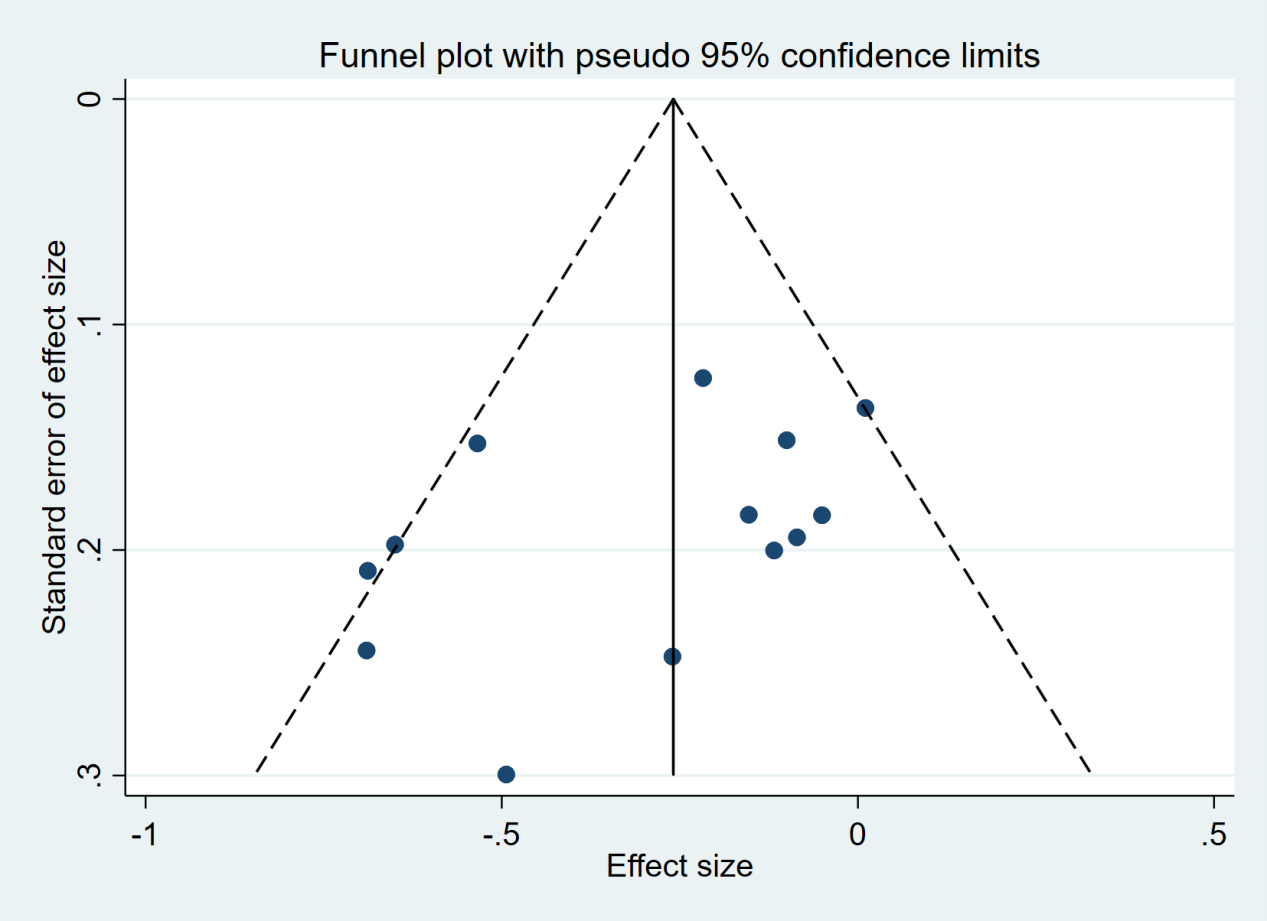
**Supplementary Figure 7** Comparison-adjusted funnel plot for Websites vs. Usual Care.

**Supplementary Table 8:** Egger test for Wearable Devices vs. Usual Care.

| Number of studies=13 | |  |  | Root MSE =1.311 | |
| --- | --- | --- | --- | --- | --- |
| **Std_Eff** | **Coef.** | **Std. Err.** | **t** | **P** | **95% CI** |
| slope | -0.11 | 0.11 | -1.06 | 0.319 | (-0.36,0.13) |
| bias | 0.11 | 0.68 | 0.16 | **0.876** | (-1.46,1.68) |


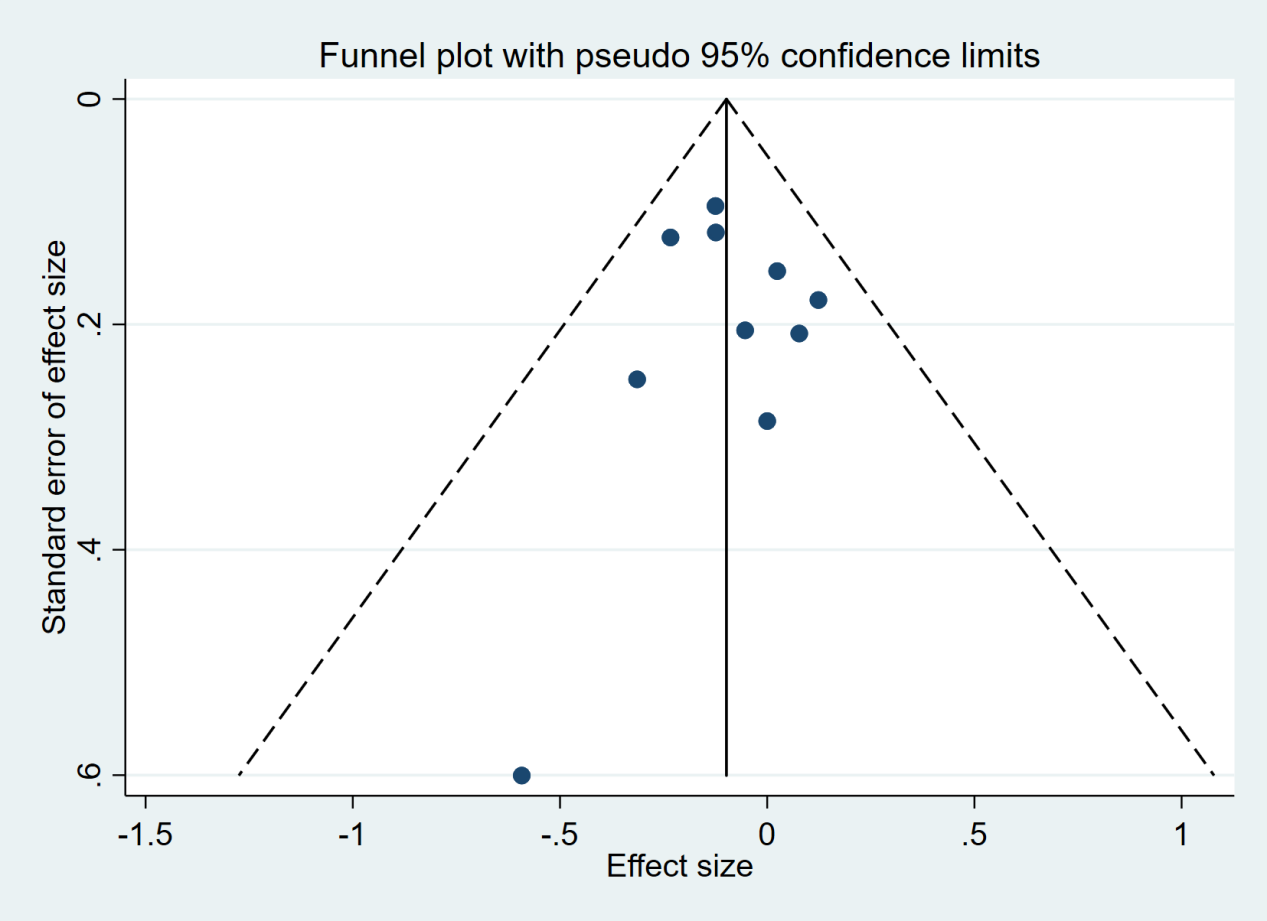
**Supplementary Figure 8:** Comparison-adjusted funnel plot for Wearable Devices vs. Usual Care.

**Supplementary Table 9:** Results of Network Meta Analysis

| **Intervention** | **Direct** | **Indirect** | **Network** | **P** |
| --- | --- | --- | --- | --- |
| CM-PC | 0.90(0.17, 1.60) | 0.00(-0.23, 0.24) | 0.09(-0.14, 0.33) | 0.022 |
| CM-SWD | 0.13(-0.37, 0.63) | 0.27(-0.05, 0.60) | 0.23(-0.03, 0.50) | **0.632** |
| CM-UC | 0.37(0.21, 0.54) | 0.89(0.31, 1.50) | 0.41(0.25, 0.57) | **0.092** |
| PC-SA | -0.50(-1.10, 0.12) | -0.07(-0.33, 0.18) | -0.13(-0.37, 0.09) | **0.202** |
| PC-SMS | -0.50(-1.20, 0.22) | -0.19(-0.52, 0.15) | -0.25(-0.54, 0.06) | **0.441** |
| PC-W | -0.40(-1.20, 0.45) | -0.04(-0.33, 0.25) | -0.07(-0.35, 0.19) | **0.442** |
| PC-UC | 0.41(0.22, 0.61) | -0.12(-0.54, 0.30) | 0.32(0.14, 0.50) | 0.024 |
| SA-W | 0.07(-0.60, 0.76) | 0.06(-0.21, 0.34) | 0.06(-0.19, 0.31) | **0.975** |
| SA-WD | -0.003(-0.91, 0.91) | 0.30(0.02, 0.59) | 0.28(0.01, 0.55) | **0.533** |
| SA-UC | 0.44(0.28, 0.61) | 0.43(-0.08, 0.95) | 0.45(0.30, 0.61) | **0.978** |
| SMS-UC | 0.53(0.25, 0.80) | 0.84(0.10, 1.60) | 0.56(0.30, 0.82) | **0.430** |
| W-UC | 0.38(0.16, 0.61) | 0.45(-0.19, 1.10) | 0.39(0.18, 0.61) | **0.850** |
| WD-UC | 0.13(-0.12, 0.39) | 0.32(-0.31, 0.96) | 0.18(-0.05, 0.40) | **0.582** |

**Supplementary Table10:** The Results of Sensitivity Analysis

| Study Omitted | | Estimate | 95%CI |
| --- | --- | --- | --- |
| NO | Author |
| 1 | Kerfoot2017 | -0.222 | (-0.256, -0.188) |
| 2 | Haak2017 | -0.222 | (-0.256, -0.189) |
| 3 | Carallo2017 | -0.215 | (-0.249, -0.182) |
| 4 | Lindberg2017 | -0.220 | (-0.253, -0.187) |
| 5 | Jing Wang2018 | -0.219 | (-0.252, -0.186) |
| 6 | Sarayani2018 | -0.221 | (-0.254, -0.188) |
| 7 | Fang2017 | -0.218 | (-0.251, -0.184) |
| 8 | Ramadas2018 | -0.221 | (-0.254, -0.188) |
| 9 | Döbler2018 | -0.215 | (-0.249, -0.182) |
| 10 | Lee2018 | -0.218 | (-0.251, -0.185) |
| 11 | Boels2019 | -0.223 | (-0.256, -0.190) |
| 12 | Wang2019 | -0.218 | (-0.252, -0.185) |
| 13 | Vinitha2019 | -0.219 | (-0.253, -0.186) |
| 14 | Yu2019 | -0.217 | (-0.250, -0.184) |
| 15 | Sun2019 | -0.218 | (-0.251, -0.184) |
| 16 | Zhai2020 | -0.219 | (-0.252, -0.186) |
| 17 | Yolanda2020 | -0.230 | (-0.264, -0.195) |
| 18 | Xu2020 | -0.219 | (-0.252, -0.185) |
| 19 | Presley2020 | -0.221 | (-0.255, -0.188) |
| 20 | Gong2020 | -0.222 | (-0.255, -0.188) |
| 21 | Lee2019 | -0.221 | (-0.254, -0.187) |
| 22 | Hilmarsdóttir2021 | -0.219 | (-0.252, -0.186) |
| 23 | Elizabeth2020 | -0.218 | (-0.251, -0.185) |
| 24 | Middleton2021 | -0.220 | (-0.254, -0.187) |
| 25 | Lim2021 | -0.218 | (-0.251, -0.184) |
| 26 | Sherifali2020 | -0.218 | (-0.252, -0.184) |
| 27 | Waller2021 | -0.224 | (-0.257, -0.190) |
| 28 | Lin2021 | -0.217 | (-0.251, -0.184) |
| 29 | Yang2021 | -0.216 | (-0.250, -0.183) |
| 30 | Turnin2021 | -0.219 | (-0.253, -0.186) |
| 31 | GLASGOW2000 | -0.222 | (-0.255, -0.189) |
| 32 | CHO2006 | -0.217 | (-0.250, -0.184) |
| 33 | Kim2007 | -0.217 | (-0.250, -0.184) |
| 34 | Marıá2009 | -0.222 | (-0.256, -0.189) |
| 35 | STONE2009 | -0.227 | (-0.261, -0.194) |
| 36 | Bujnowska-Fedak2010 | -0.221 | (-0.254, -0.187) |
| 37 | Cho2011 | -0.219 | (-0.252, -0.186) |
| 38 | Tildesley2011 | -0.218 | (-0.252, -0.185) |
| 39 | Goodarzi2012 | -0.218 | (-0.251, -0.185) |
| 40 | McMahon2012 | -0.220 | (-0.253, -0.187) |
| 40 | McMahon2012 | -0.219 | (-0.252, -0.186) |
| 40 | McMahon2012 | -0.220 | (-0.253, -0.187) |
| 41 | Williams2012 | -0.217 | (-0.250, -0.184) |
| 42 | Orsama2013 | -0.218 | (-0.251, -0.185) |
| 43 | Heisler2014 | -0.221 | (-0.254, -0.188) |
| 44 | Steventon2014 | -0.222 | (-0.256, -0.189) |
| 45 | Wakefield2014 | -0.221 | (-0.255, -0.188) |
| 46 | Waki2014 | -0.218 | (-0.251, -0.185) |
| 47 | Zhou2014 | -0.216 | (-0.249, -0.183) |
| 48 | Holmen2014 | -0.220 | (-0.253, -0.186) |
| 49 | Nicolucci2015 | -0.218 | (-0.251, -0.184) |
| 50 | Bentley2016 | -0.219 | (-0.252, -0.186) |
| 50 | Bentley2016 | -0.219 | (-0.252, -0.186) |
| 51 | Crowley2016 | -0.219 | (-0.252, -0.185) |
| 52 | Lim2016 | -0.218 | (-0.251, -0.184) |
| 53 | Odnoletkova2016 | -0.224 | (-0.258, -0.190) |
| 54 | Trief2016 | -0.222 | (-0.255, -0.189) |
| 55 | Wild2016 | -0.214 | (-0.248, -0.181) |
| 56 | Agboola2016 | -0.222 | (-0.256, -0.189) |
| 57 | Fortmann2017 | -0.217 | (-0.250, -0.184) |
| 58 | Dario2017 | -0.223 | (-0.256, -0.190) |
| 59 | Kempf2017 | -0.216 | (-0.249, -0.182) |
| 60 | Young2017 | -0.221 | (-0.255, -0.188) |
| 60 | Young2017 | -0.222 | (-0.256, -0.189) |
| 61 | Alghafri2018 | -0.221 | (-0.255, -0.188) |
| 62 | Kooiman2018 | -0.219 | (-0.252, -0.186) |
| 63 | Kusnanto 2019 | -0.218 | (-0.252, -0.185) |
| 64 | Storch,2019 | -0.215 | (-0.249, -0.182) |
| 65 | Huang2019 | -0.219 | (-0.253, -0.186) |
| 66 | Kim2019 | -0.222 | (-0.255, -0.189) |
| 66 | Kim2019 | -0.218 | (-0.251, -0.185) |
| 67 | Millan-Ferro2020 | -0.220 | (-0.254, -0.187) |
| 68 | Shahsavari2020 | -0.216 | (-0.249, -0.183) |
| 69 | Sokolovska2020 | -0.219 | (-0.252, -0.186) |
| 70 | Asante2020 | -0.217 | (-0.250, -0.184) |
| 71 | Furler2020 | -0.219 | (-0.253, -0.186) |
| 72 | Ku2020 | -0.218 | (-0.251, -0.185) |
| 73 | Wang2020 | -0.219 | (-0.252, -0.186) |
| 74 | Yang2020 | -0.217 | (-0.250, -0.183) |
| 75 | Farmer2021 | -0.238 | (-0.272, -0.203) |
| 76 | Hu2021 | -0.215 | (-0.248, -0.182) |
| 77 | Li2021 | -0.221 | (-0.254, -0.188) |
| 78 | Patnaik2021 | -0.222 | (-0.255, -0.188) |
| 79 | Guo2021 | -0.218 | (-0.251, -0.185) |
| 80 | Kumar2021 | -0.217 | (-0.251, -0.183) |
| 81 | Lyu2021 | -0.220 | (-0.254, -0.187) |
| 82 | Poonprapai2022 | -0.216 | (-0.249, -0.183) |
| 83 | Yeojin2022 | -0.216 | (-0.250, -0.183) |
| 84 | Kasar2022 | -0.219 | (-0.252, -0.185) |
| 85 | Timurtas2022 | -0.220 | (-0.253, -0.187) |
| 86 | Xia2022 | -0.211 | (-0.244, -0.178) |
| 87 | Brodnik2014 | -0.218 | (-0.251, -0.185) |
| 88 | Rosas2017 | -0.218 | (-0.252, -0.185) |
| Combined |  | -0.219 | (-0.253, -0.186) |
